# Unicentric Castleman disease in the left adrenal region: a case report and literature review

**DOI:** 10.3389/fonc.2025.1638855

**Published:** 2025-09-15

**Authors:** Zhen Wang, Yong Shi, Yun Chen, Zhongle Xu, Li Zheng, Yanbin Zhang, Junhua Xi

**Affiliations:** ^1^ Department of Urology, The Second People’s Hospital of Hefei, Hefei Hospital Affiliated to Anhui Medical University, Hefei, China; ^2^ The Fifth Clinical School of Medicine, Anhui Medical University, Hefei, China; ^3^ Department of Pathology, The Second People’s Hospital of Hefeim. Hefei Hospital Affiliated to Anhui Medical University, Hefei, China

**Keywords:** Castleman disease, unicentric Castleman disease, adrenal tumor, hyalinevascular type, retroperitoneal lesion, case report

## Abstract

**Background:**

Castleman disease (CD) is a rare lymphoproliferative disorder that can present either as unicentric (UCD) or multicentric (MCD), with distinct clinical and pathologic features. Involvement of the adrenal region is extremely uncommon, often mimicking more common adrenal tumors such as pheochromocytoma. This report describes a patient with a solitary retroperitoneal lesion in the left adrenal region who was ultimately diagnosed with unicentric Castleman disease (hyaline vascular type).

**Case presentation:**

A 44-year-old female was admitted for surgical management of a left retroperitoneal lesion initially suspected to be an adrenal tumor, based on imaging studies (including CT and PET-CT). Laboratory tests ruled out pheochromocytoma or endocrine hyperfunction. During robotic-assisted laparoscopic surgery, a well-defined 5-cm lesion adjacent to the left adrenal gland was resected along with regional lymph nodes. Pathological examination confirmed Castleman disease of the hyaline vascular subtype with fibrosis and calcification. Postoperative recovery was uneventful, and the patient was discharged with an excellent prognosis.

**Conclusions:**

Castleman disease manifesting in the adrenal region is exceedingly rare and may be easily mistaken for an adrenal neoplasm, especially when imaging reveals a hypervascular retroperitoneal lesion with calcification. This case underscores the importance of including Castleman disease in the differential diagnosis of indeterminate adrenal-region tumors with normal endocrine function. Complete surgical excision typically confers an excellent prognosis for unicentric disease.

## 1 Introduction

Castleman disease (CD) is a rare lymphoproliferative disorder marked by characteristic pathological changes in lymph nodes. First reported by Benjamin Castleman in 1954, CD is classified into two clinical forms: unicentric Castleman disease (UCD), involving a single lymph node or region, and multicentric Castleman disease (MCD), which presents with systemic manifestations, multiple lymph node regions, and potential associations with HHV-8 infection, POEMS syndrome, or idiopathic multicentric Castleman disease (iMCD) (1, 2). Histologically, CD is categorized into three main subtypes: hyaline vascular (HV), plasma cell (PC), and a mixed type (3).

CD most commonly affects the mediastinal (70%), cervical, and retroperitoneal lymph nodes, whereas involvement of the adrenal region is extremely rare (4, 5). In the few published cases, Castleman disease in the adrenal region often mimics pheochromocytoma or other adrenal tumors, as imaging typically shows a well-enhancing retroperitoneal lesion (6, 7). The hyaline vascular type, which accounts for approximately 90% of UCD cases, is characterized by small hyalinized follicles and interfollicular vascular proliferation (8).

We report a case of a 44-year-old woman with a left adrenal-region lesion pathologically confirmed as unicentric Castleman disease, hyaline vascular subtype, with fibrosis and calcification. Relevant literature is reviewed to highlight diagnostic challenges and management strategies for this rare condition.

## 2 Case report

### 2.1 Patient presentation and workup

A 44-year-old woman was admitted for evaluation of a left retroperitoneal lesion, incidentally detected during gastrointestinal investigations for chronic reflux symptoms. She initially presented with a three-year history of acid reflux and heartburn, without fever, cough, chest tightness, or abdominal pain. Initial abdominal CT scans revealed a solid, well-demarcated lesion (5.3 cm) near the left adrenal gland, with focal calcifications. Her past medical history was notable for reflux esophagitis and a hiatal hernia; there was no chronic hypertension, diabetes, or hepatitis.

**Figure 1:**
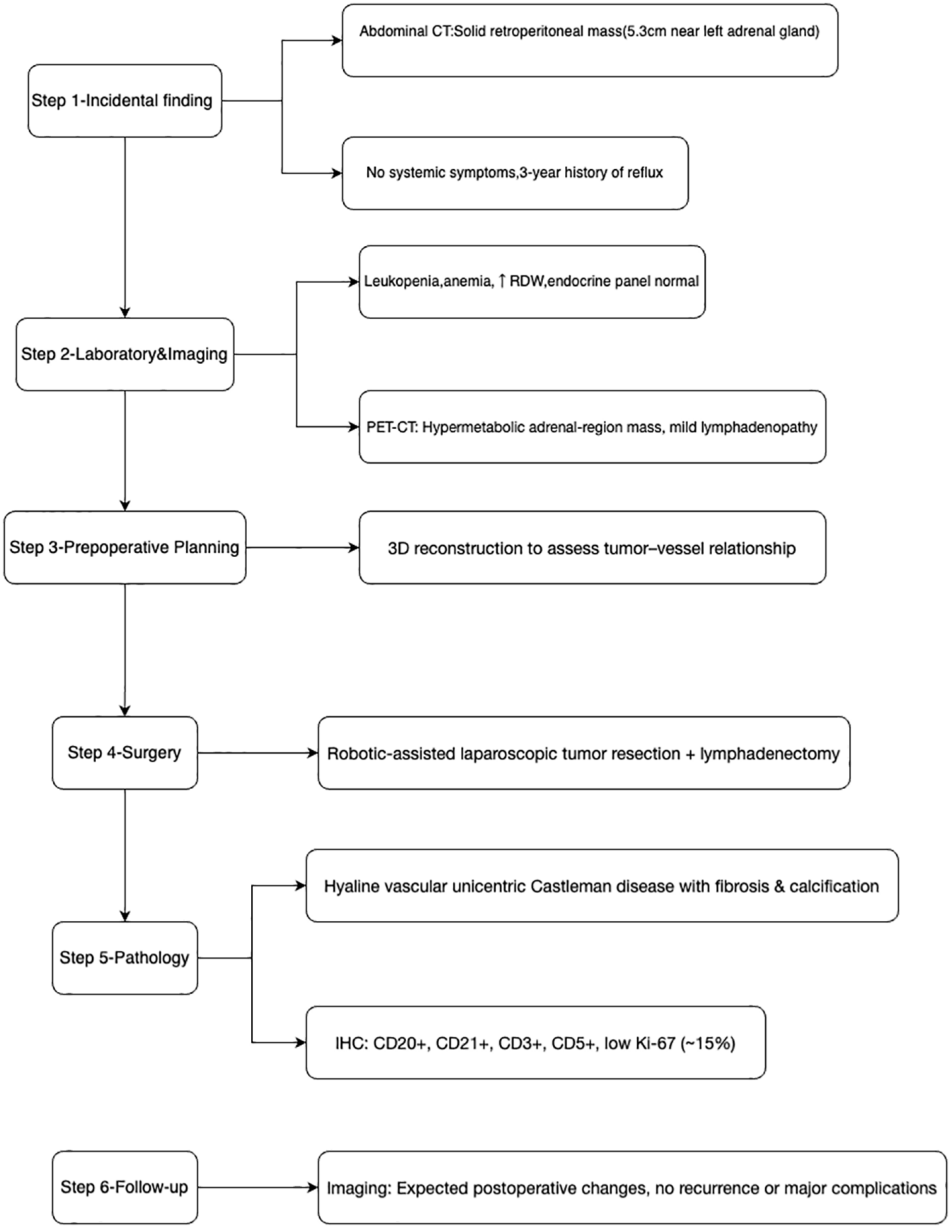
Timeline and diagnostic–therapeutic workflow for the patient.

Physical examination revealed normal vital signs (T 36.6°C, P 72/min, R 18/min, BP 105/62 mmHg) with mild epigastric tenderness but no palpable lesions or lymphadenopathy. The patient was initially admitted to the general surgery department for management of her reflux symptoms, but was subsequently transferred to urology after the adrenal lesion was identified. The overall diagnostic and therapeutic pathway for this patient is summarized in **Figure 1**.

#### 2.1.1 Laboratory and endocrine findings

Laboratory and endocrine results are summarized in **Table 1**. The patient exhibited marked leukopenia, anemia, and an elevated red cell distribution width, indicating anemia with anisocytosis. Endocrine evaluation revealed no evidence of adrenal hormonal hypersecretion, effectively excluding pheochromocytoma and other functional adrenal tumors. Immunologic assessment showed normal CRP and ESR, no significant elevation of IL-6, and negative serology for HIV and HHV-8, supporting a diagnosis of non-viral hyaline vascular unicentric Castleman disease. All other laboratory parameters were within reference ranges.

**Table 1:** Summary of laboratory and endocrine findings.

Category	Parameter	Result	Reference range	Interpretation
Hematology	White blood cell count	2.87 ×10^9^/L	3.50–9.50 ×10^9^/L	↓ Leukopenia
Hemoglobin	87.0 g/L	115–150 g/L	↓ Anemia	
Hematocrit	28.7%	35.0–45.0%	↓ Low	
Mean corpuscular volume (MCV)	75.9 fL	80–100 fL	↓ Slightly low	
Mean corpuscular hemoglobin (MCH)	22.9 pg	27–34 pg	Low	
Mean corpuscular hemoglobin concentration (MCHC)	302 g/L	320–360 g/L	Low	
Red cell distribution width (RDW-CV)	16.7%	11.5–14.5%	↑ Elevated (anisocytosis)	
Neutrophil count	1.76 ×10^9^/L (61.7%)	2.0–7.0 ×10^9^/L	Slightly low	
Lymphocyte count	0.72 ×10^9^/L (24.9%)	1.0–3.0 ×10^9^/L	Low	
Endocrine	Adrenal hormonal profile	—	—	No evidence of hypersecretion; pheochromocytoma excluded
Immunology	CRP, ESR	Within normal limits	—	No inflammation
Immunoglobulin G (IgG)	23 g/L	—	Normal	
Interleukin-6 (IL-6)	—	—	Not significantly elevated	
HIV, HHV-8	Negative	—	Supports non-viral UCD	

Other laboratory parameters were within reference ranges.

#### 2.1.2 Imaging studies

**Figure 2:**
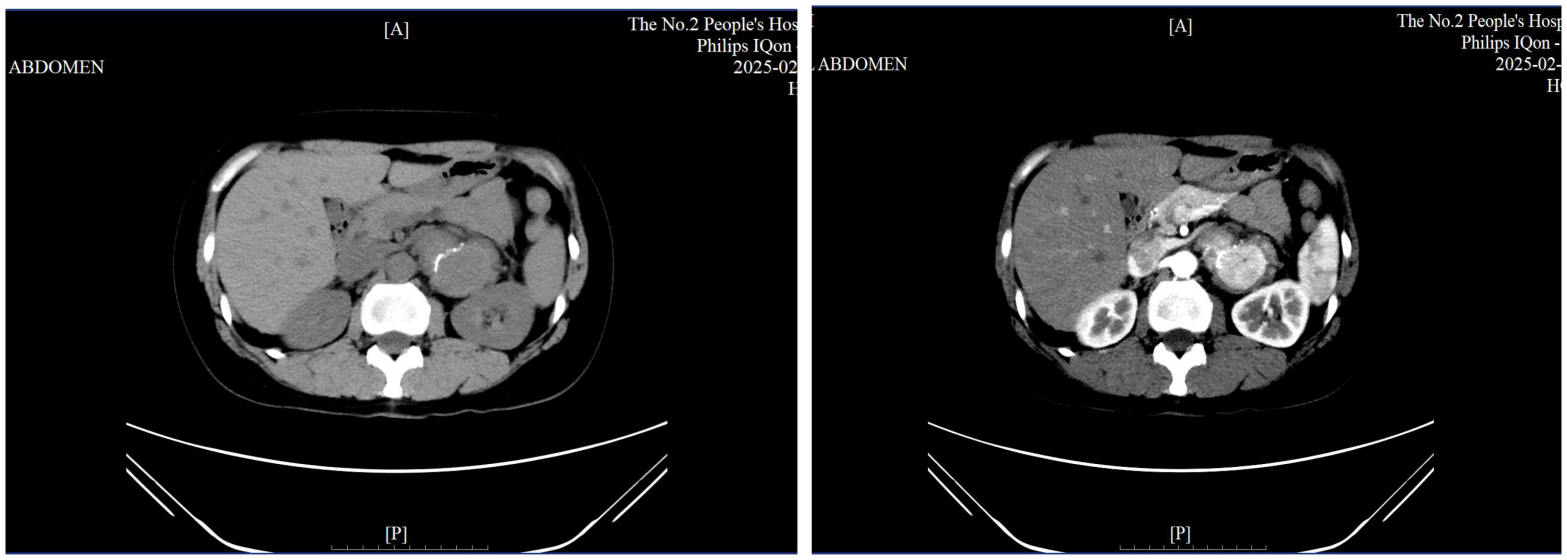
Contrast-enhanced CT scan showing a well-defined 5.3 cm lesion in the left adrenal region with calcification.

**Figure 3:**
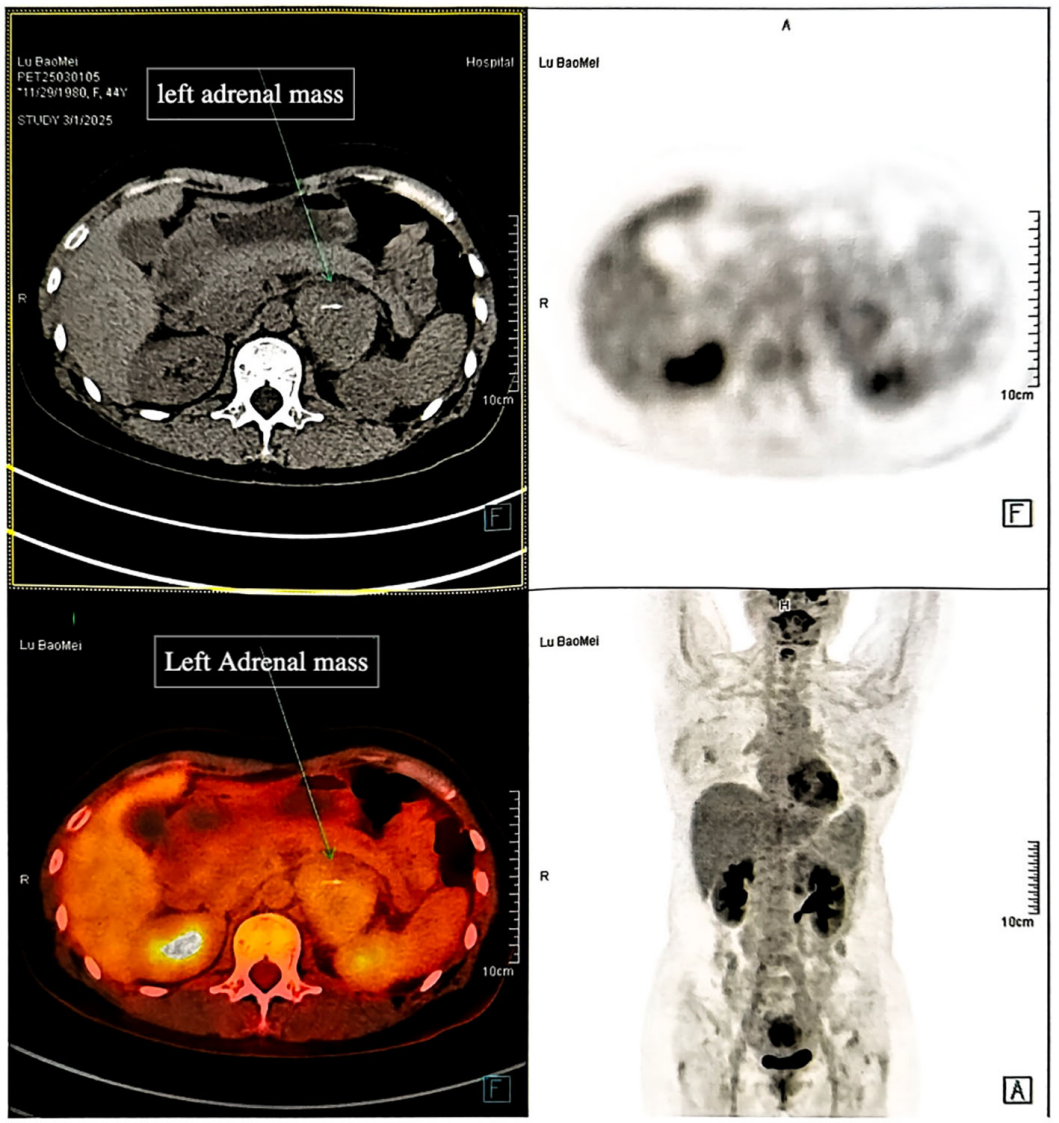
PET-CT scan demonstrating increased FDG uptake in the left adrenal region lesion.

- Chest CT: Revealed a left retroperitoneal lesion with partial coronary artery calcification.
- Contrast-enhanced abdominal CT: A well-defined, 5.3 cm soft-tissue lesion in the left adrenal area, showing moderate enhancement in arterial and venous phases (**Figure 2**), with calcification and adjacent lymph node enlargement. The radiologist initially suggested pheochromocytoma as the most likely diagnosis, with recommendations to exclude vascular lesions, adrenocortical carcinoma, or neurogenic tumors.
- PET-CT: The lesion exhibited increased metabolic activity suggesting a hypervascular or hypermetabolic process (**Figure 3**). Several regional lymph nodes also showed mildly increased tracer uptake.
- Upper gastrointestinal contrast study and gastroscopy: Confirmed reflux esophagitis (LA-B) and chronic non-atrophic gastritis with erosions, but no significant structural abnormalities.

### 2.2 Surgical Intervention

**Figure 4:**
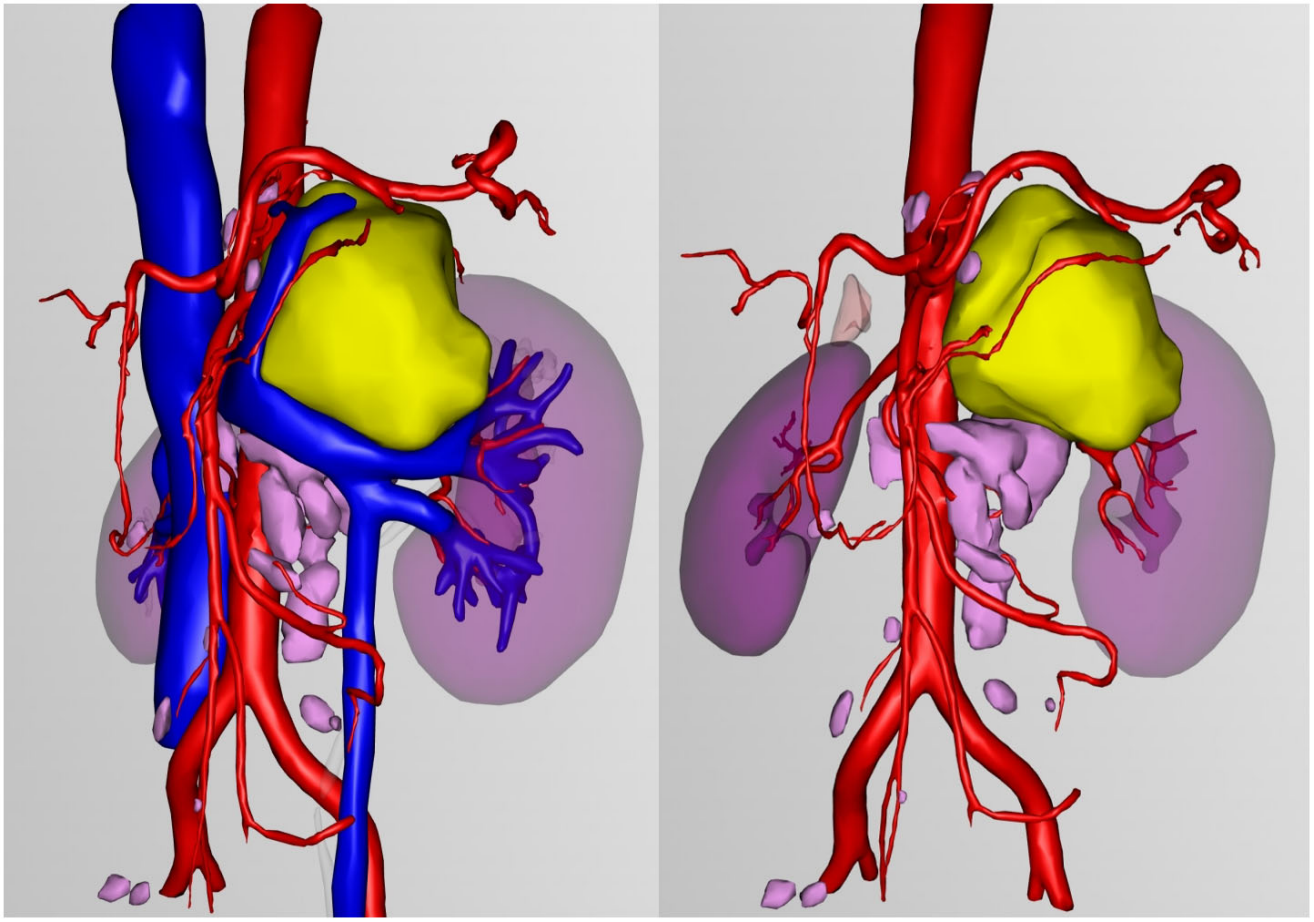
Three-dimensional reconstruction: Yellow represents retroperitoneal tumors, pink represents lymph nodes.

Due to the unclear boundary between the retroperitoneal tumor and the renal arteries, preoperative three-dimensional reconstruction was performed (**Figure 4**). On March 11, 2025, Da Vinci robot-assisted retroperitoneal tumor resection and lymph node dissection were carried out.

**Figure 5:**
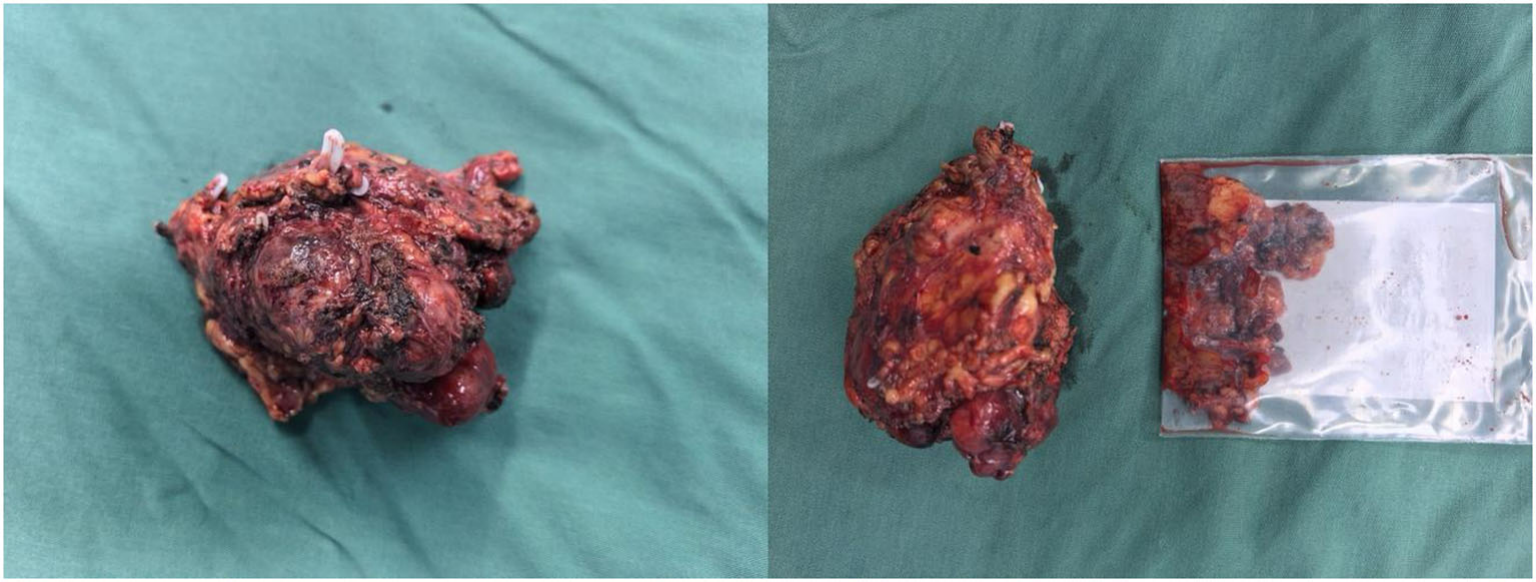
Gross specimen of unicentric Castleman disease located in the left adrenal region.

**Figure 6:**
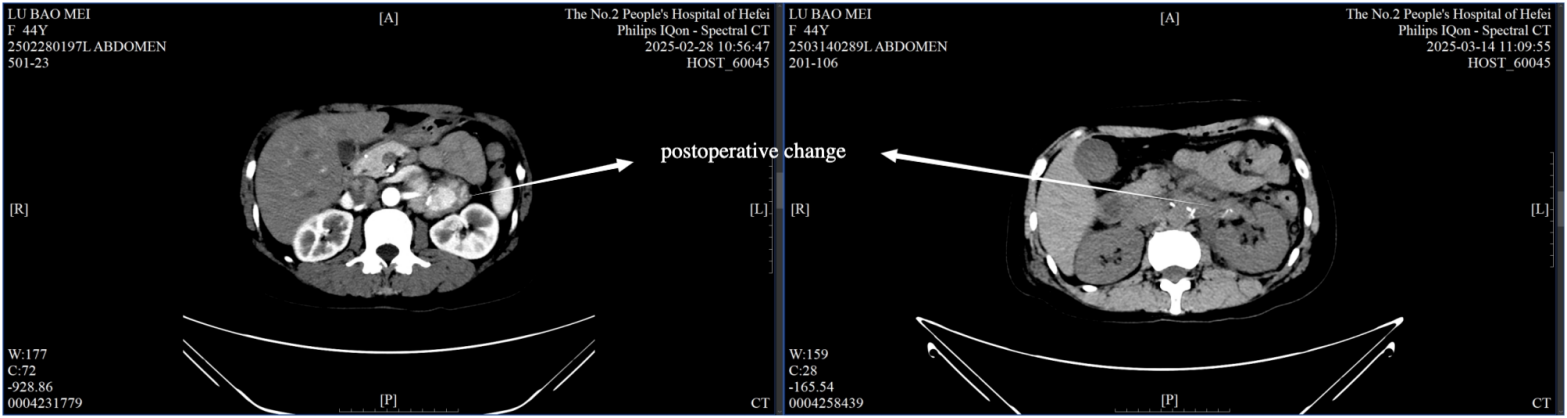
Postoperative non-enhanced CT scan.

Intraoperatively, a firm, 5 cm × 4 cm lesion was found closely abutting the left adrenal gland and partially attached to the surrounding fat and musculature. The tumor caused marked compression of the kidney and renal vessels, and multiple enlarged lymph nodes were noted beneath the renal vein. The lesion (**Figure 5**) was completely mobilized after careful dissection of the renal vessels and adrenal central vein, with hemostasis achieved using Hem-O-Lock clips. Retroperitoneal fat was cleared, and 21 enlarged lymph nodes were excised. Postoperative non-enhanced CT (**Figure 6**) demonstrated expected surgical changes without evidence of residual lesion or recurrence.

### 2.3 Pathological findings

- Gross specimen: A gray-red to gray-yellow irregular tissue measuring 8.5 cm × 5.5 cm × 5.0 cm, with intact capsule and focal bone-like consistency.
- Microscopic examination: Lymph node structure with atrophic follicles, interfollicular vascular proliferation, and characteristic “onion-skin” arrangement, consistent with the hyaline vascular subtype of Castleman disease. Notable fibrosis and calcification were present.
- Final diagnosis: Unicentric Castleman disease, hyaline vascular type, with fibrosis and calcification.
- The 21 resected retroperitoneal lymph nodes showed reactive hyperplasia without evidence of malignancy.

**Figure 7:**
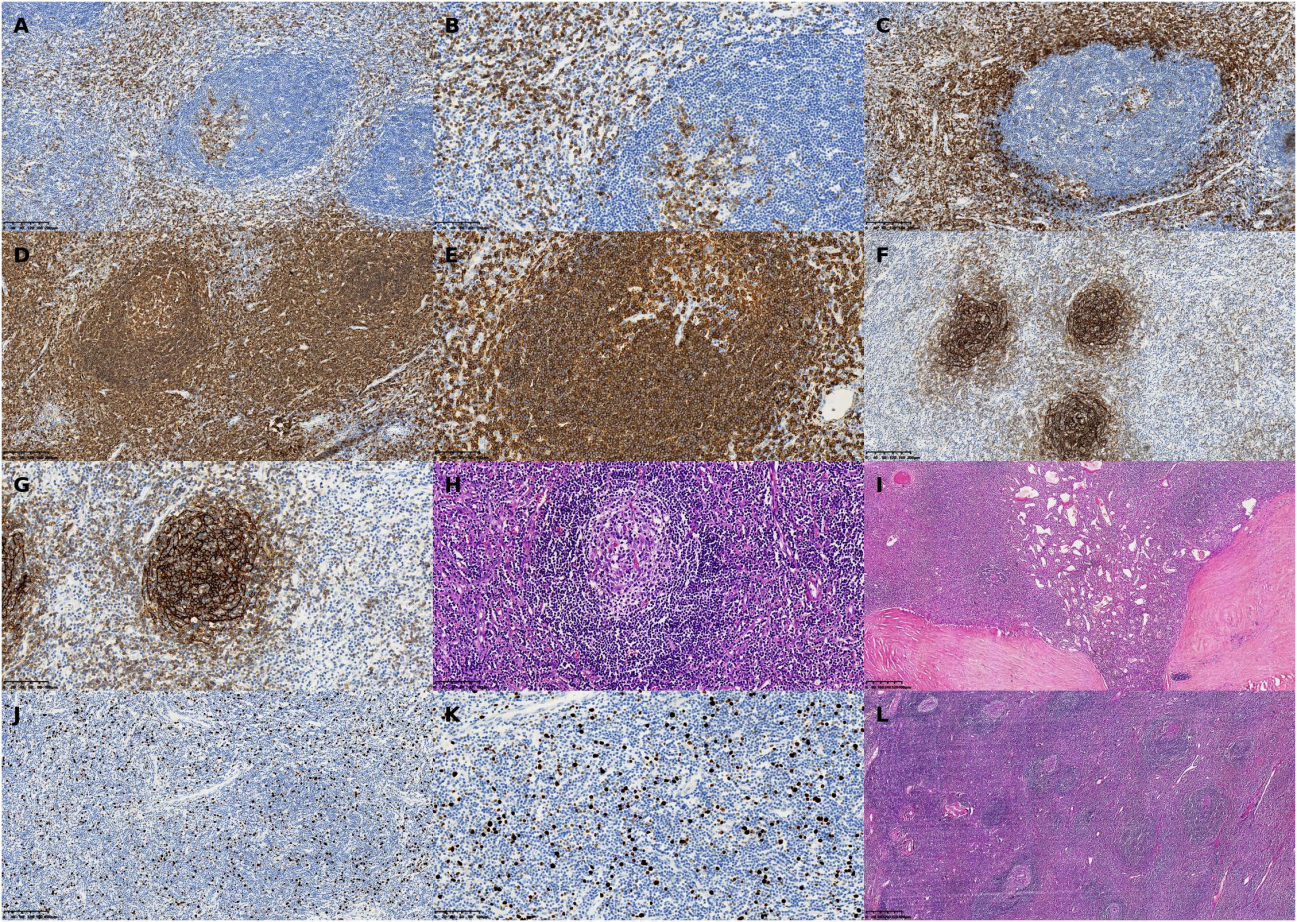
Representative histopathologic and immunohistochemical findings in a patient with unicentric Castleman disease (hyaline vascular type) involving the left adrenal region. **(A)** CD3 immunohistochemical stain, ×40: Low-power view showing nodal architecture with fibrotic stroma and increased vascularity. **(B)** CD5 immunohistochemical stain, ×40: Demonstrates follicular hyperplasia with prominent capillary networks in the interfollicular areas. **(C)** CD5 immunohistochemical stain, ×200: High magnification revealing the characteristic “onion-skin” concentric layering of lymphocytes around atrophic germinal centers. **(D)** CD3, ×200: Immunostaining showing strong membranous expression of T-cell marker CD3 in the paracortical zone. **(E)** CD5, ×100: Positive staining along T-cell membranes in the interfollicular areas. **(F)** CD5, ×200: High-power view further demonstrating diffuse CD5 positivity, highlighting the T-cell-rich zones. **(G)** CD20, ×100: B-cell marker showing follicular positivity consistent with reactive germinal centers. **(H)** H&E stain, ×200: Enhanced expression in germinal center B cells, with well-defined follicular boundaries. **(I)** H&E stain, ×100: Follicular dendritic cell (FDC) meshwork evident within germinal centers. **(J)** CD21, ×200: Reticular mesh pattern of FDCs confirmed at higher magnification. **(K)** Ki-67, ×100: Immunostaining showing scattered nuclear positivity with an estimated proliferation index of ~15%. **(L)** H&E stain, ×200: Higher magnification validating low proliferative activity, supporting the diagnosis of a benign lymphoproliferative process.

Given the histologic similarity of Castleman disease to other reactive lymphadenopathies, differential diagnoses such as rheumatoid arthritis (9), systemic lupus erythematosus (SLE) (10), and tuberculous lymphadenitis (11) were considered. Relevant autoantibody screening, including anti-adrenocortical antibodies, was performed and returned negative. Histopathologic examination did not reveal caseating granulomas or acid-fast bacilli, effectively ruling out adrenal tuberculosis (**Figure 7**).

### 2.4 Postoperative course and follow-up

The postoperative course was uneventful, with only mild incisional discomfort that gradually resolved with symptomatic management. Follow-up chest and abdominal CT on March 14 (**Figure 6**) revealed expected postoperative changes in the left adrenal region, including minimal pleural effusion and a small localized fluid collection, without any evidence of recurrence or complications requiring further intervention. The patient was discharged on March 19, 2025, with instructions for rest, nutritional support, and ongoing management of gastric symptoms.

## 3 Discussion

### 3.1 Rarity and diagnostic challenges

Although Castleman disease commonly involves thoracic or abdominal lymph nodes, occurrence in or near the adrenal gland is extremely rare. A review of the literature identified fewer than 20 reported cases of adrenal or peri-adrenal Castleman disease (12, 13). Previous studies (e.g., 6, 14) have reported that adrenal Castleman disease typically appears as a well-defined, hyperenhancing lesion on CT, often mimicking pheochromocytoma (9, 14). This was consistent with our patient’s presentation, in whom normal endocrine evaluations were accompanied by a suspicious hypermetabolic lesion with calcification, leading to a presumptive diagnosis of adrenal tumor.

The diagnostic challenge is compounded by the absence of specific clinical manifestations. In our case, the reflux symptoms were entirely unrelated to the retroperitoneal lesion, which was detected incidentally, which was discovered incidentally. This finding is consistent with previous reports indicating that adrenal Castleman disease is often asymptomatic and detected during investigations for unrelated conditions (15, 16).

### 3.2 Imaging insights

Imaging characteristics of adrenal Castleman disease can be misleading. On CT, these lesions typically appear as well-defined, homogeneously enhancing lesions, sometimes with calcifications—features that overlap with pheochromocytoma, adrenal adenoma, and other adrenal neoplasms (16, 17). In our case, the presence of calcification within the lesion is noteworthy, as this feature has been reported in only a subset of Castleman disease cases and may reflect the chronic nature of the process with resultant dystrophic calcification (18).

Contrast-enhanced CT may assist in characterizing these lesions, as Castleman disease typically demonstrates homogeneous enhancement in both arterial and venous phases (19). However, this enhancement pattern is not specific and may also be observed in other hypervascular adrenal lesions. The presence of enlarged regional lymph nodes, as seen in our patient, may offer an additional clue to the lymphoproliferative nature of the disease.

### 3.3 Histopathology

A definitive diagnosis is established through histopathological examination. The hyaline vascular (HV) subtype, which our patient exhibited, is the most common type in unicentric disease, accounting for approximately 90% of UCD cases (20, 21). It features regressed germinal centers, increased follicular dendritic cells, and excessive angiogenesis with the characteristic “onion-skin” arrangement of lymphocytes around the follicles (22).

The immunohistochemical profile in our case (CD20+, CD21+, CD3+, CD5+, CD38 partially+, CD138 few+) is consistent with previous reports of hyaline vascular Castleman disease (23). The low Ki-67 proliferation index (15%) further supports the benign nature of this entity. The presence of fibrosis and calcification in our specimen represents a less commonly reported feature that may reflect the chronic nature of the disease process.

Plasma cell (PC) and mixed variants are more often encountered in multicentric presentations and may associate with systemic symptoms (e.g., fever, anemia, organomegaly) (24). These variants typically demonstrate sheets of mature plasma cells in the interfollicular areas and may be associated with elevated interleukin-6 (IL-6) levels (25).

### 3.4 Treatment and prognosis

Surgical resection is curative for unicentric Castleman disease, particularly the hyaline vascular (HV) type, and remains the gold standard of treatment (26, 27). Recently published international diagnostic and treatment guidelines for unicentric Castleman disease recommend complete surgical excision as the first-line management (28). Complete excision, as achieved in our patient, typically results in resolution of any associated symptoms and prevents recurrence. The minimally invasive approach of robotic-assisted laparoscopy, as employed in our case, offers advantages over open surgery, including reduced postoperative pain, shorter hospitalization, and faster recovery (29). The patient underwent preoperative three-dimensional reconstruction, which can guide precise intraoperative procedures and help minimize damage to surrounding organs and major blood vessels.

Recurrence after complete excision of UCD is rare, with reported rates of less than 5% (30). Long-term follow-up with periodic imaging is recommended; however, the optimal surveillance protocol remains undefined because of the rarity of the disease.

In contrast, multicentric Castleman disease (MCD) generally requires systemic therapy, including monoclonal antibodies targeting interleukin-6 (siltuximab) or rituximab, particularly in HHV-8–associated or idiopathic MCD (iMCD) cases (31, 32). The excellent postoperative course in our patient aligns with the favorable prognosis typically observed in UCD.

## 4 Conclusion

Castleman disease arising in the left adrenal region is an extremely rare condition that may mimic a functional adrenal tumor. This case underscores several key clinical considerations: (1) in patients with normal endocrine findings but persistent imaging suspicion, Castleman disease should be included in the differential diagnosis for adrenal region lesions; (2) the presence of calcification within a well-defined, enhancing retroperitoneal lesion may raise suspicion for Castleman disease, although this finding is not pathognomonic; (3) complete surgical resection serves as both a diagnostic and therapeutic approach for unicentric disease.

Surgical resection, guided by preoperative imaging and meticulous intraoperative assessment, remains the mainstay for definitive diagnosis and treatment in unicentric hyaline vascular cases. Complete excision generally confers an excellent prognosis with a minimal risk of recurrence. This case contributes to the limited body of literature on adrenal Castleman disease and emphasizes the importance of considering this rare condition in the differential diagnosis of adrenal region lesions.

## Data Availability

The raw data supporting the conclusions of this article will be made available by the authors, without undue reservation.
